# Unique Sensitization Patterns to Allergen Components in a Crustacean‐Allergic Australian Cohort

**DOI:** 10.1002/clt2.70120

**Published:** 2026-06-11

**Authors:** Elecia Johnston, Sahel Heidari, Shaymaviswanathan Karnaneedi, Diamond Hira, Sandip Kamath, Andreas Ludwig Lopata

**Affiliations:** ^1^ Molecular Allergy Research Laboratory College of Public Health Medical and Vet Sciences James Cook University Townsville Queensland Australia; ^2^ Australian Institute of Tropical Health and Medicine James Cook University Townsville Queensland Australia; ^3^ Centre for Food and Allergy Research Murdoch Children's Research Institute Melbourne Victoria Australia; ^4^ Allergy Medical Centre Mundingburra Queensland Australia; ^5^ MacroArray Diagnostics Vienna Austria; ^6^ Tropical Futures Institute James Cook University Singapore Singapore Singapore

To the Editor,

Crustacean allergy affects 1%–3% of the global population, with shrimp being a common trigger. Tropomyosin (TM) is reported to be the primary allergen in up to 80% of cases, but other allergens such as arginine kinase (AK), myosin light chain (MLC), and haemocyanin may also contribute to allergic reactions. Despite the widespread prevalence of crustacean allergies, the diagnostic accuracy of whole extracts versus single components remains underexplored in Oceania [1, 2, 3, 4]. This is particularly important given the variety of crustacean species, including crabs and lobsters, each containing allergens that may differ in sequence homology and in their relative abundance across species.

This study reports allergic sensitisation patterns in 54 individuals with confirmed crustacean allergy (Supporting Information S1: Table S1), who exhibited clinical symptoms and tested positive in skin prick tests (SPT) and/or ImmunoCAP assays (Table 1). Specific IgE sensitisation was assessed using the ALEX2 multiplex IgE test (Macro Array Diagnostics), which includes four crustacean whole extracts: shrimp mix (*Litopenaeus setiferus*, *Farfantepenaeus aztecus*, *Farfantepenaeus duorarum*), *Pandalus borealis* (Pan b), *Chionoecetes* spp. (Chi spp.), and *Homarus gammarus* (Hom g), along with five single allergen components: Pen m 1, Pen m 2, Pen m 3, Pen m 4, and Cra c 6. Subjects testing negative to all crustacean allergens on the array were further analysed using immunoblotting against *Penaeus monodon* extract and purified haemocyanin, a shrimp species widely consumed in Australia and globally. Details of the protocol are provided in the Supplementary Methods.

**TABLE 1:** Demographics and diagnostic results of 54 subjects.

Type of patients	Paediatric % (*N*)	33.3% (18)
Age	Adult % (*n*)	66.6% (36)
	Median (min‐max)	28.5 (9–73)
Mite ALEX	Pos % (*n*)	85.2% (46)
	Neg % (*n*)	14.8% (8)
HDM immunotherapy	Yes % (*n*)	29.6% (16)
	No % (*n*)	70.3% (38)
Gender	Female % (*n*)	55.5% (30)
	Male % (*n*)	44.4% (24)
Crustacean immunocap (prawn, crab)	Pos % (*n*)	77.7% (42)
	Neg % (*n*)	16.6% (9)
	ND % (*n*)	5.5% (3)
	Mean IgE (min‐max) (KU/L)	3.86 (0‐> 100)
Crustacean SPT (raw/cooked prawn and crab)	Pos % (*n*)	90.7% (49)
	Neg % (*n*)	1.8% (1)
	ND % (*n*)	7.4% (4)
	Mean wheal size (min‐max) (mm)	4.48 (0–20)

The subjects displayed a wide array of IgE binding patterns, highlighting the complex nature of sensitization within this population. TM sensitisation was demonstrated in 40.7% of the cohort, followed by troponin C (TnC) (26%), AK (22%), sarcoplasmic calcium‐binding protein (SCP) (13%), and MLC (4%) (Table 1; Supporting Information S1: Figure S1A). Notably, 45% did not show any binding to TM, which is generally considered to be the major allergen in crustacean allergy. Among these TM‐negative subjects, 53.1% were instead sensitised to other allergens. Importantly, while 20% of the cohort were sensitised to multiple allergens, the majority (52%) were monosensitized to one of the five allergens tested, with the highest binding observed for TnC, followed by AK, SCP, and MLC (Table 1, Supporting Information S1: Figure S1B). Similar high reactivity to TnC was also observed in other studies from tropical regions in Hong Kong and Thailand [5]. IgE levels against *P. monodon* TM (Pen m 1) showed strong correlations with TMs from *Anisakis simplex* (Ani s 3), *Dermatophagoides pteronyssinus* (Der p 10), *Blomia tropicalis* (Blo t 10), and *Periplaneta americana* (Per a 7), with *R* ^2^ values ranging from 0.89 to 0.96 (Figure 1A, Supporting Information S1: Table S2A). These findings are consistent with previously reported cross‐reactivity among invertebrate tropomyosins [6, 7]. In contrast, IgE responses to arginine kinase (Pen m 2) were only weakly correlated with homologues from *Dermatophagoides pteronyssinus* (Der p 20) and *Blattella germanica* (Bla g 9), with *R* ^2^ values of 0.57 and 0.73, respectively (Supporting Information S1: Table S2B, Figure S2).

**FIGURE 1:**
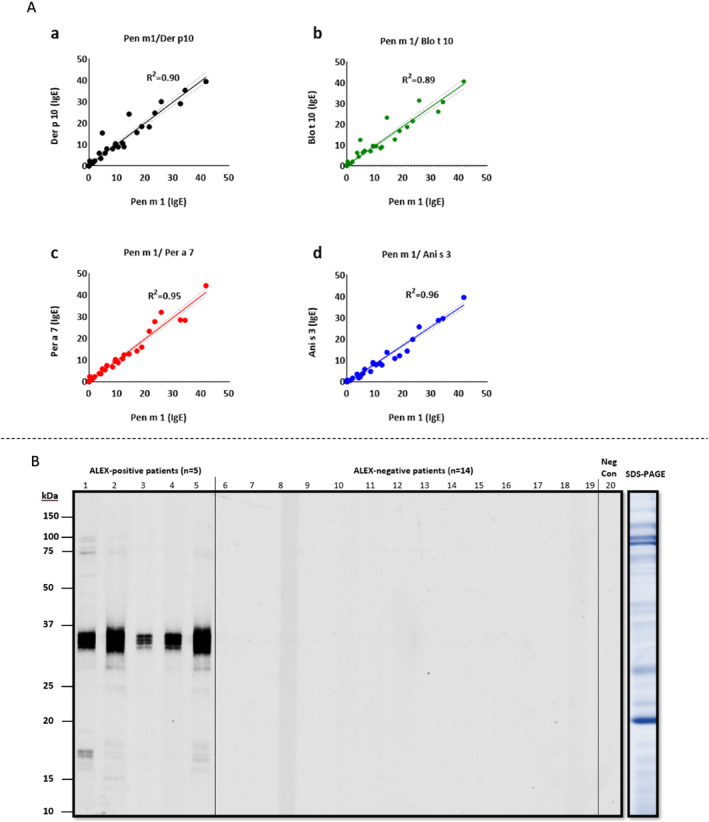
IgE binding to tropomyosin across species and IgE reactivity to *Penaeus monodon* protein extract. (A) Linear regression of IgE binding to Pen m 1 and homologous tropomyosins: (a) Der p 10, (b) Blo t 10, (c) Per a 7, (d) Ani s 3. *R* ^2^ values above 0.7 indicate strong positive correlations. (B) Immunoblotting using sera from ALEX [2]‐positive (*n* = 5) and ALEX [2]‐negative (*n* = 14) patients. Lanes one to five show ALEX [2]‐positive sera, lanes 6–19 are ALEX [2]‐negative, and lane 20 is the negative control. SDS‐PAGE of protein separation is shown on the right; molecular weights (kDa) are indicated on the left.

To better understand the clinical relevance of sensitisation profiles, we examined clinically recorded symptom patterns. Most subjects (52%) experienced gastrointestinal symptoms, followed by 36% with cutaneous reactions (Supporting Information S1: Figure S3A). The clinical significance of other allergens is evident, as TM‐negative subjects also displayed symptoms, with elevated IgE levels not directly correlating with symptom severity (Supporting Information S1: Figure S3C). This suggests that other allergens may contribute to symptom manifestation in addition to tropomyosin.

To assess whether additional allergens are necessary for an accurate serological diagnosis, we evaluated the diagnostic sensitivity of TM combined with other single allergens and whole allergen extracts. Combining single allergens resulted in a sensitivity of 72%, with only a marginal 2% increase when including whole extracts, suggesting minimal improvement in diagnostic accuracy (Supporting Information S1: Table S3). While previous studies suggested that haemocyanin might enhance diagnostic sensitivity [8, 9], immunoblot analysis of 14 negative individuals revealed no IgE binding to purified haemocyanin (Supporting Information S1: Figure S4). Additionally, no IgE binding was detected to any proteins in the *P. monodon* extract, which may indicate that missing shrimp allergens are not the sole explanation for the negative IgE results in subjects with a clear positive history of crustacean allergy (Figure 1B). However, the potential absence of relevant allergens in both the ALEX panel and our natural extracts cannot be excluded and may have contributed to the observed lack of IgE reactivity in some individuals. It is also important to note that other crab and lobster allergens were not assessed. This may also reflect the limited protein repertoire in the extracts used for testing and purification of natural allergens, which could result in underrepresentation of allergens relevant for some subjects. While PBS‐based extraction protocols are reproducible and widely used, they may underrepresent low‐abundance or less soluble proteins. Future studies using alternative extraction strategies could help capture a broader range of clinically relevant crustacean allergens for routine testing.

In conclusion, our study emphasises the significant heterogeneity of IgE responses to shrimp allergens and suggests that sensitization to various crustacean species, especially in tropical regions, plays a crucial role in shaping unique sensitization patterns. These findings highlight the limitations of current extract‐based diagnostics, underscoring the necessity of incorporating single‐component allergens for improved sensitivity. Furthermore, expanding diagnostic panels to include allergens from other crustaceans, such as crab and lobster, will not only enhance diagnostic accuracy but also pave the way for more personalised and effective management strategies for allergic individuals.

## Author Contributions

**Elecia Johnston:** investigation, formal analysis, writing – original draft, writing – review and editing. **Sahel Heidari:** writing – original draft, writing – review and editing, investigation, formal analysis. **Shaymaviswanathan Karnaneedi:** investigation, data curation. **Diamond Hira:** data curation, resources. **Sandip Kamath:** resources, data curation. **Andreas Ludwig Lopata:** conceptualisation, supervision, methodology, writing – review and editing, project administration.

## Funding

This study was supported by Centre for Food and Allergy Research and James Cook University.

## Conflicts of Interest

The authors declare no conflicts of interest.

## Supporting information


Supporting Information S1

## Data Availability

The data that support the findings of this study are available on request from the corresponding author. The data are not publicly available due to privacy or ethical restrictions.

## References

[1] C. Gamez , S. Sanchez‐Garcia , M. D. Ibanez , et al., “Tropomyosin IgE‐Positive Results Are a Good Predictor of Shrimp Allergy,” Allergy 66, no. 10 (October 2011): 1375–1383, 10.1111/j.1398-9995.2011.02663.x. 21651567

[2] M. A. Faber , M. Pascal , O. El Kharbouchi , et al., “Shellfish Allergens: Tropomyosin and Beyond,” Allergy 72, no. 6 (June 2017): 842–848, 10.1111/all.13115. 28027402

[3] B. B. Su , W. Blackmon , C. Xu , et al., “Diagnosis and Management of Shrimp Allergy,” Front Allergy 5 (2024): 1456999, 10.3389/falgy.2024.1456999. 39493746 PMC11527777

[4] R. Asero , G. Mistrello , S. Amato , et al., “Shrimp Allergy in Italian Adults: A Multicenter Study Showing a High Prevalence of Sensitivity to Novel High Molecular Weight Allergens,” International Archives of Allergy and Immunology 157, no. 1 (2012): 3–10, 10.1159/000324470. 21894023

[5] C. Y. Y. Wai , N. Y. H. Leung , A. S. Y. Leung , et al., “Comprehending the Allergen Repertoire of Shrimp for Precision Molecular Diagnosis of Shrimp Allergy,” Allergy 77, no. 10 (October 2022): 3041–3051, 10.1111/all.15370. 35567339 PMC9795902

[6] S. Dramburg , C. Hilger , A. F. Santos , et al., “EAACI Molecular Allergology User's Guide 2.0,” Pediatric Allergy & Immunology 34, no. Suppl 28 (March 2023): e13854, 10.1111/pai.13854. 37186333

[7] S. D. Kamath , M. Bublin , K. Kitamura , T. Matsui , K. Ito , and A. L. Lopata , “Cross‐Reactive Epitopes and Their Role in Food Allergy,” Journal of Allergy and Clinical Immunology 151, no. 5 (May 2023): 1178–1190, 10.1016/j.jaci.2022.12.827. 36932025

[8] J. Grilo , U. Vollmann , M. Aumayr , G. J. Sturm , and B. Bohle , “Tropomyosin Is No Accurate Marker Allergen for Diagnosis of Shrimp Allergy in Central Europe,” Allergy 77, no. 6 (June 2022): 1921–1923, 10.1111/all.15290. 35322447 PMC9321988

[9] O. Mendoza‐Porras , S. Kamath , J. O. Harris , et al., “Resolving Hemocyanin Isoform Complexity in Haemolymph of Black Tiger Shrimp Penaeus Monodon—Implications in Aquaculture, Medicine and Food Safety,” Journal of Proteomics 218 (April 2020): 103689, 10.1016/j.jprot.2020.103689. 32088355

