# Supplementary material for: Unique Sensitization Patterns to Allergen Components in a Crustacean‐Allergic Australian Cohort
Source: Clin Transl Allergy. 2026 Jun 11;16(6):e70120. doi: 10.1002/clt2.70120 (PMC13255744; doi:10.1002/clt2.70120)
Supplement: Supplementary file 1 — Supporting Information S1 [file CLT2-16-e70120-s001.docx]

**Supplementary/Supporting Tables and Figures**

**Material and methods**

**Shrimp Allergy Subjects**

As part of Australia's largest seafood allergy cohort study, we recruited 93 seafood-allergic patients, including 54 with confirmed shellfish allergy and positive SPTs and/or ImmunoCAP results (f23, f24, f80, f320). The cohort included paediatric (<18 years) and adult participants (Table S1). Blood samples were collected in EDTA tubes, centrifuged at 3,000 rpm for 10 min at 4°C, and stored at -80°C. Ethical approval was granted by the James Cook University Ethics Committee (H4313, H6829), with all participants providing written informed consent. Clinical symptoms were classified per U.S. food allergy guidelines [1] into cutaneous, ocular, gastrointestinal, respiratory, and cardiovascular categories.

**Clinical Diagnostic Tests**

SPTs were conducted using commercial crab and shrimp extracts (ALK-Abelló, Madrid, Spain), with histamine (10 mg/mL) as a positive control and saline as a negative control. A wheal >3 mm after 15 minutes was considered positive. Specific IgE (sIgE) levels were measured via ImmunoCAP (Thermo Fisher, USA) and ALEX_2_ (Macro Array Diagnostics, Vienna, Austria), with positivity defined as ≥0.35 kU/L. The ALEX2 assay detects IgE against 117 allergen extracts and 178 purified components, including five single allergen components from shrimps —TM, AK, MLC, SCP (*Penaeus monodon)*, and TnC (*Crangon crangon*)—and four whole crustacean extracts —Chi spp., Hom g, shrimp mix, and Pan b—following the manufacturer’s protocol.

**Protein and hemocyanin Extraction and Quantification**

Raw black tiger prawn extract was prepared following a previously described method [2]. Briefly, Muscle tissue of black tiger prawn (*Penaeus monodon*) was blended with PBS (pH 7.2), mixed overnight at 4°C, centrifuged (13,000 rpm, 4°C, 20 min), filter-sterilized, and stored at −80°C. Total protein was quantified using the BCA Assay (Thermo Scientific, Waltham, MA, USA) following the manufacturer’s protocol.

Hemocyanin was purified from pooled haemolymph plasma from *P. monodon* after removal of haemocytes through ultracentrifugation. Purity was established by SDS-gel electrophoresis and FASP MS analysis as previously described [3].

**SDS-PAGE and Immunoblotting**

Based on a previously reported method [4], *P.monodon* extract (5 µg) and purified hemocyanin (0.5-1 µg) were separated on 12% Bis-Tris gels, run at 170 V for 1 hour, and stained with Coomassie Brilliant Blue. SDS-PAGE and immunoblotting were performed under denaturing and reduction conditions. For Immunoblot analysis, purified HC was transferred to a nitrocellulose membrane (Trans-Blot® SD, BioRad), blocked with 0.1% casein (Sigma), and detected using polyclonal anti-HC (1:50,000, in-house) and anti-sheep IgG (1:15,000, Thermo Scientific). For serum IgE binding analysis, 1 µg purified protein and 5 µg tiger prawn extract were separated, transferred, and blocked under the same conditions. The membrane was then incubated overnight with serum (1:15 in PBST + 0.2× casein) and incubate with anti-human IgE (1:1000, Santa-Cruz) and anti-mouse IgG (1:10,000, Li-Cor). Detection was performed using the Odyssey® CLx Imaging System.

**Results**

**Supplementary Table 1:** Demographics and diagnostic results of 54 patients.


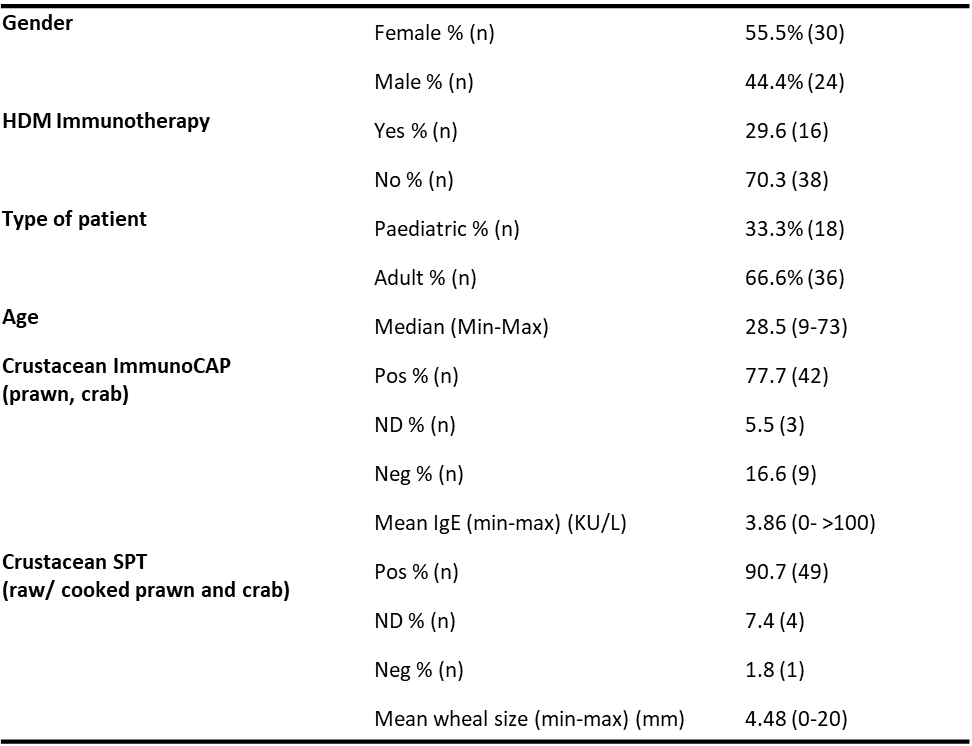


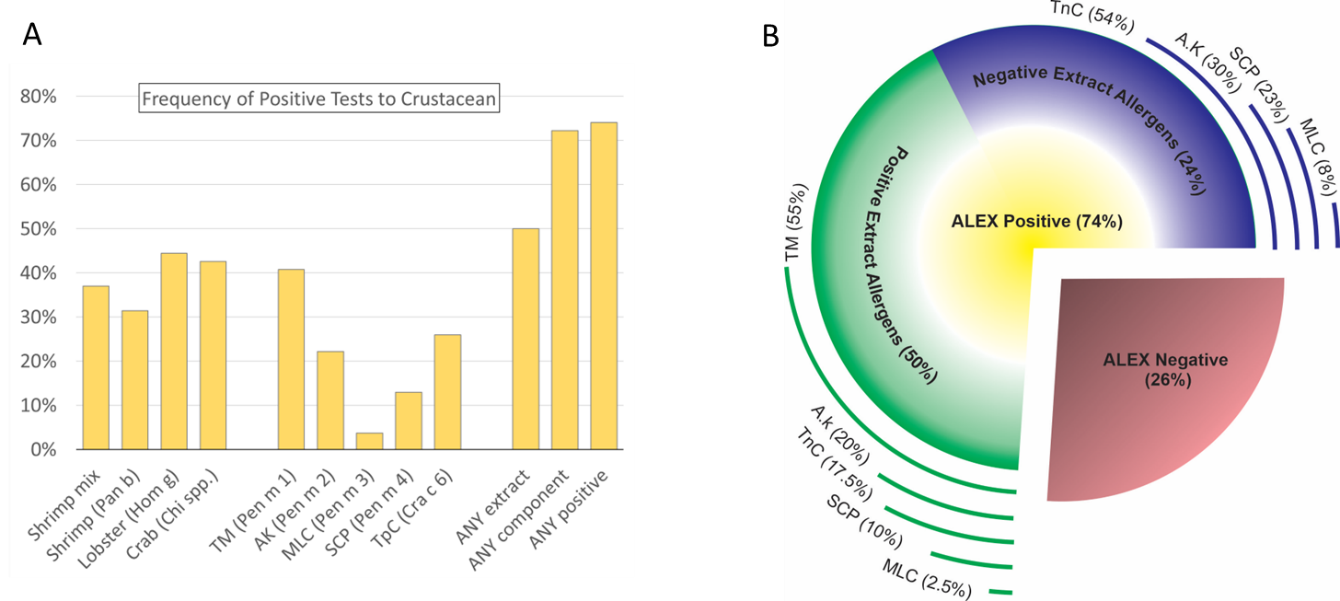


**Supplementary Figure 1: A)** Frequency of positive tests to crustacean extracts and single component allergens on the ALEX_2_ array. **B)** Distribution of patient sensitization patterns tested with ALEX_2_. Green: ALEX-positive patients to whole extracts or components; Blue: negative to any extracts but positive to components; Red: ALEX-negative patients. A specific IgE result of ≥0.35 kU/L is considered positive. TM: Tropomyosin, AK: Arginine kinase, MLC: Myosin light chain, SCP: Sarcoplasmic calcium-binding protein, TnC: Troponin C.


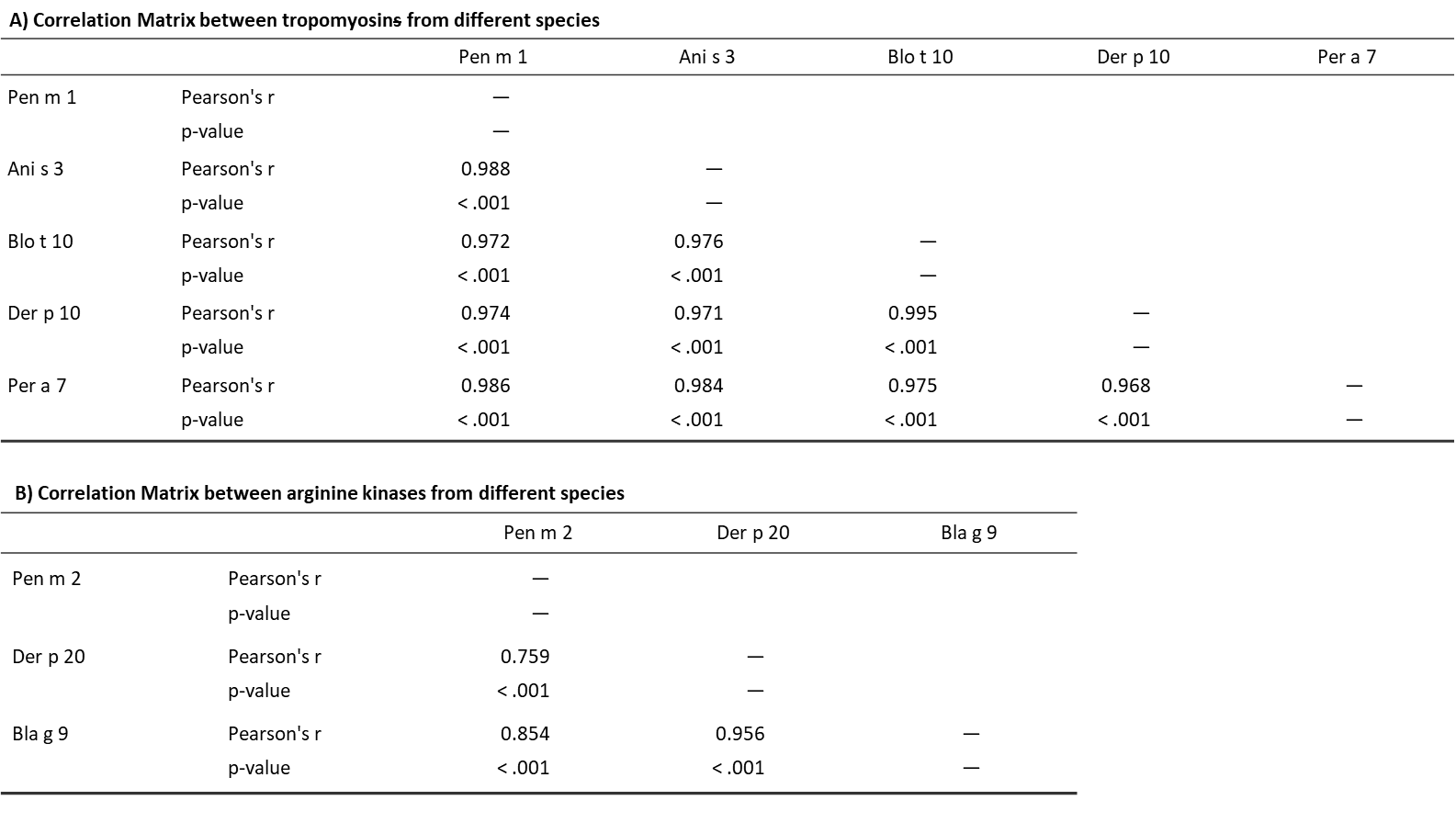
**Supplementary Table 2:** Amino acid sequence comparison between different tropomyosins and arginine kinase by Pearson correlation matrix. **A)** Pearson coefficients for comparing the positive results between Pen m 1, Ani s 3, Blo t 10, Der p 10, and Per a 7. **B)** Pearson coefficients for comparing the positive results between Pen m 2, Der p 20, and Bla g 9. P-value less than 0.05 is considered significant.

**Supplementary Figure 2: Relationship Between the Level of IgE Binding to Arginine Kinase (AK) in Different Species.** Linear Regression of IgE binding to AK: **A)** Pen m 1 and Der p 20. **B)** Pen m 1 and Blo g 9. R-squared values above 0.7 are considered indicative of a strong positive relationship.


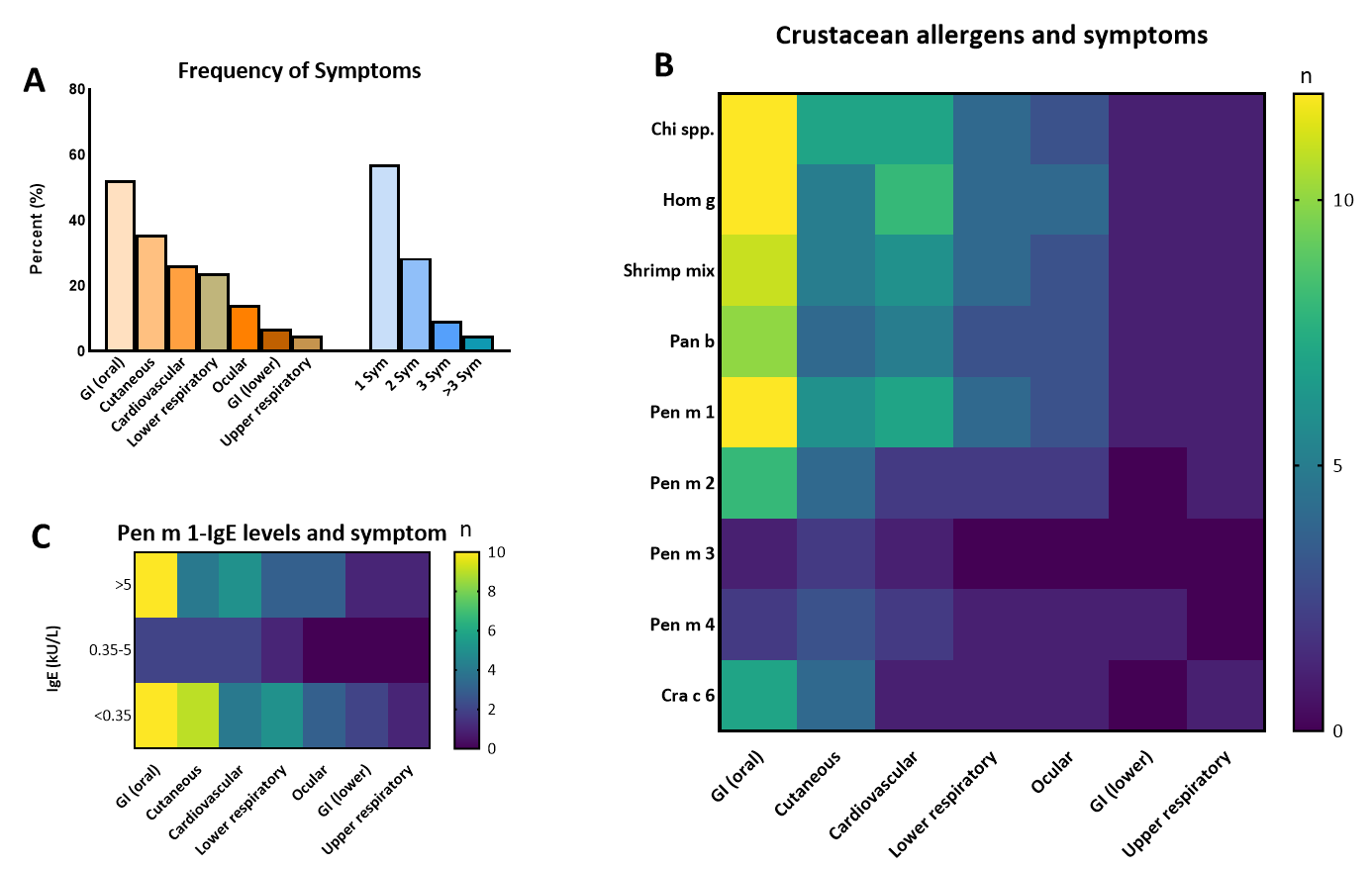


**Supplementary Figure 3: Clinical symptoms of crustacean-allergic patients (n=42)**. **A)** Frequency of symptoms and occurrence of individual and multiple symptoms, **B)** Relation between ALEX_2_ shellfish allergen tests and symptoms, **C)** Distribution of clinical symptoms across Pen m 1-specific IgE levels. GI: Gastrointestinal.

**Supplementary Table 3:** Diagnostic sensitivity of tropomyosin (Pen m 1) in combination with the other single allergen components and whole protein extracts.


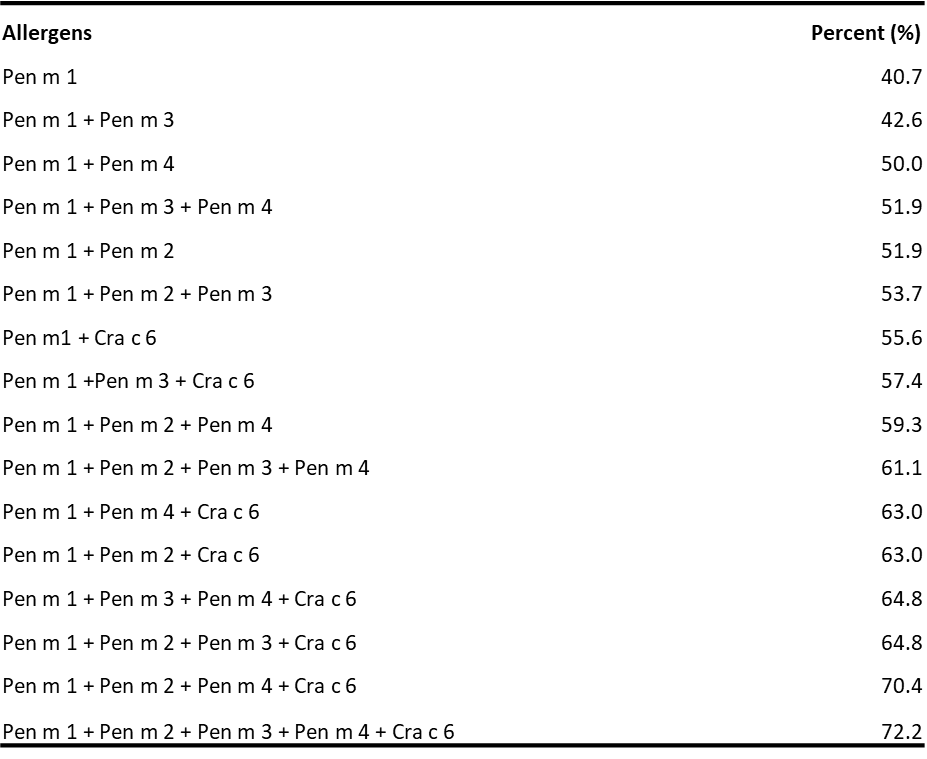


**
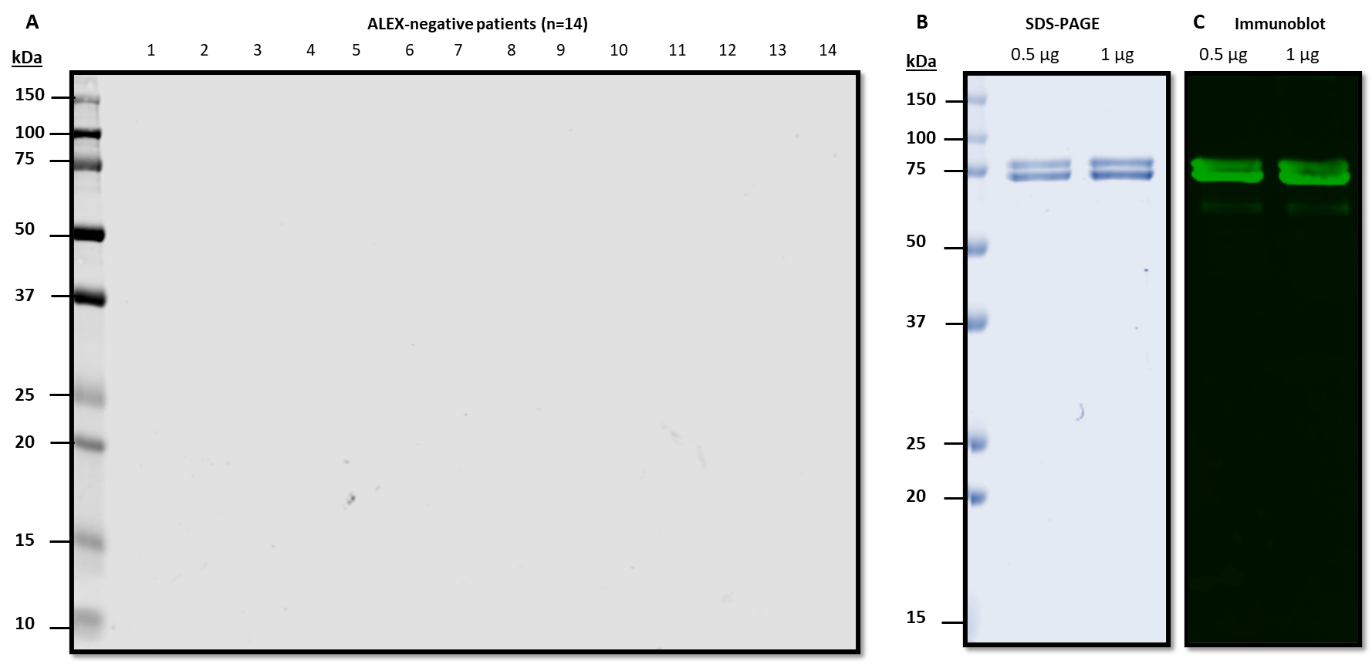
**

**Supplementary Figure 4: Immunoblot and SDS-PAGE analysis of natural black tiger shrimp hemocyanin.** **A)** IgE reactivity to natural black tiger shrimp hemocyanin protein (1 µg). Immunoblotting was performed using sera from patients (n=14) who tested negative for all crustacean component allergens and crustacean extracts. **B)** SDS-PAGE analysis of natural hemocyanin at two different concentrations (0.5 µg and 1 µg). **C)** Immunoblot analysis using in-house and polyclonal anti-hemocyanin antibodies at different concentrations (0.5 µg and 1 µg).

**Reference:**

1. Panel NI-SE, Boyce JA, Assa'ad A, Burks AW, Jones SM, Sampson HA, et al. Guidelines for the diagnosis and management of food allergy in the United States: report of the NIAID-sponsored expert panel. J Allergy Clin Immunol. 2010;126(6 Suppl):S1-58. doi: 10.1016/j.jaci.2010.10.007.

2. Abramovitch JB, Kamath S, Varese N, Zubrinich C, Lopata AL, O'Hehir RE, et al. IgE Reactivity of Blue Swimmer Crab (Portunus pelagicus) Tropomyosin, Por p 1, and Other Allergens; Cross-Reactivity with Black Tiger Prawn and Effects of Heating. PLoS One. 2013;8(6):e67487. doi: 10.1371/journal.pone.0067487.

3. Mendoza-Porras O, Kamath S, Harris JO, Colgrave ML, Huerlimann R, Lopata AL, et al. Resolving hemocyanin isoform complexity in haemolymph of black tiger shrimp Penaeus monodon - implications in aquaculture, medicine and food safety. J Proteomics. 2020;218:103689. doi: 10.1016/j.jprot.2020.103689.

4. Nugraha R, Ruethers T, Taki AC, Johnston EB, Karnaneedi S, Kamath SD, et al. Recombinant Tropomyosin from the Pacific Oyster (Crassostrea gigas) for Better Diagnosis. Foods. 2022;11(3). doi: 10.3390/foods11030404.
